# The impact of social cohesion and risk communication on excess mortality due to COVID-19 in 213 countries: a retrospective analysis

**DOI:** 10.1186/s12889-024-19076-7

**Published:** 2024-06-14

**Authors:** Ricardo Eccard da Silva, Maria Rita Carvalho Garbi Novaes, Cesar de Oliveira, Dirce Bellezi Guilhem

**Affiliations:** 1 Brazilian Health Regulatory Agency - Anvisa, Setor de Indústrias, Trecho 5, Área Especial 57, Brasília, 71205-050 DF Brazil; 2 Faculty of Health Sciences, University of Brasília – UnB, Campos Univ. Darcy Ribeiro, Asa Norte, Brasília, 70910-900 DF Brazil; 3 Department of Epidemiology & Public Health, University College London (UCL), 1-19 Torrington Place, London, WC1E 6BT UK

**Keywords:** Covid-19, Pandemics, Excess mortality, Social cohesion, Health communication

## Abstract

**Background:**

Tools for assessing a country’s capacity in the face of public health emergencies must be reviewed, as they were not predictive of the COVID-19 pandemic. Social cohesion and risk communication, which are related to trust in government and trust in others, may have influenced adherence to government measures and mortality rates due to COVID-19.

**Objective:**

To analyse the association between indicators of social cohesion and risk communication and COVID-19 outcomes in 213 countries.

**Results:**

Social cohesion and risk communication, in their dimensions (public trust in politicians, trust in others, social safety nets, and equal distribution of resources index), were associated with lower excess mortality due to COVID-19. The number of COVID-19-related disorder events and government transparency were associated with higher excess mortality due to COVID-19. The lower the percentage of unemployed people, the higher the excess mortality due to COVID-19. Most of the social cohesion and risk communication variables were associated with better vaccination indicators, except for social capital and engaged society, which had no statistically significant association. The greater the gender equality, the better the vaccination indicators, such as the number of people who received all doses.

**Conclusion:**

Public trust in politicians, trust in others, equal distribution of resources and government that cares about the most vulnerable, starting with the implementation of programs, such as cash transfers and combating food insecurity, were factors that reduced the excess mortality due to COVID-19. Countries, especially those with limited resources and marked by social, economic, and health inequalities, must invest in strengthening social cohesion and risk communication, which are robust strategies to better cope with future pandemics.

**Supplementary Information:**

The online version contains supplementary material available at 10.1186/s12889-024-19076-7.

## Background

Risk communication is an important component of disaster risk management, as it influences risk perceptions and actions in the face of these events. The credibility of the information source impacts the decisions of individuals who receive the information. For example, in Japan, the level of trust in government and other official communications was severely tested in the Fukushima nuclear accident. Effective risk communication requires trust among involved parties, along with proper training and planning for execution [1].

Social cohesion refers to the extent of social connectedness and solidarity between different community groups within a society, and the level of trust and connectedness between individuals and across community groups. Social cohesion is a critical resource for disaster recovery planning and an important component of the pre-disaster, acute, post-disaster, and recovery phases. Community engagement and strong social networks are instrumental in identifying priorities and solutions that are more likely to be appropriate, lasting, and supported by the affected community. The COVID-19 pandemic has impacted social cohesion at various levels, and the strength of social cohesion before the pandemic is likely a strong indicator of recovery. The division between the public and government is of concern during a disaster and is largely in the hands of the government. This relationship is often cited as a key to social cohesion, with central concepts being inclusive leadership, legitimacy of authority, shared identity, and common goals [2].

A study that evaluated The Global Health Security Index (GHS), which measures a country’s capacity to prepare for and control epidemics and pandemics, pointed out that the inclusion of political, governance and social cohesion aspects in the GHS may benefit the ability to assess preparedness, detection and response, as these variables were significantly associated with excess mortality in countries over the first five hundred days of the COVID-19 pandemic. Additionally, in that same study, factors such as geographic connectivity and proportion of the elderly population increased the risk profile of countries and can be used to map the most vulnerable countries [3].

The Joint External Evaluation (JEE), which is a tool launched by the World Health Organization to evaluate a country’s capacity for ensuring health security threats, does not take into account indicators related to health inequalities. These indicators can be important in assessing countries’ preparedness and response capacity [4]. Especially at the beginning of the COVID-19 pandemic, when political factors and vulnerabilities not foreseen in the GHS and JEE influenced response actions and decreased the chances of success. Thus, the GHS and JEE need to be revised to assess the inclusion of new indicators that can better estimate the response capacity of countries in the face of public health threats [5].

A previous study found that the performance of countries’ health systems, as assessed by the GHS, during the COVID-19 pandemic, did not align with the outcomes of COVID-19 such as mortality. In other words, countries that were well-classified in the GHS had high mortality rates [6]. Investment in human resources is an important aspect of pandemic preparedness. The results of a study showed that countries with the highest scores in this regard, as measured by the GHS instruments and the *International Health Regulation State Party Self-Assessment Annual Report* (IHR-SPAR), had fewer COVID-19 cases and deaths in the first eight weeks of the pandemic [7].

The best-managed and most effective vaccination process is related to the highest levels of governance indicators. To better prepare for upcoming global health threats, countries need to increase investments in health infrastructure and rational use of resources and promote good public governance. Public trust in government interfered with decision-making for adherence to vaccination programs, which impacted the number of cases and deaths caused by COVID-19 [8].

Communication of reliable and timely information, for example, about the risks and vulnerabilities involved in the scenario, is an important aspect of increasing public trust in government, even when information is still limited. Investments in risk communication and societal engagement strategies are needed to increase adherence to government measures and increase trust [9].

The analysis of variables related to social cohesion and risk communication may contribute to improving the prediction of the available instruments concerning a country’s capacity to cope with emergencies, because, currently, these instruments do not include these variables. Therefore, the objective of this study was to analyse the association between indicators of social cohesion and risk communication and COVID-19 outcomes in 213 countries.

## Methods

### Study design

This was a documentary and descriptive study. It was a correlation analysis of secondary data in the public domain. The total number of countries analysed was 213. The complete list of countries is presented in supplementary material 1.

### Data source

The databases used were: (i) GovData360 – World Bank; (ii) Data Futures Platform (United Nations Development Programme – UNDP); (iii) United Nations E-Government Knowledgebase; (iv) The Economist; (v) Oxford COVID-19 Government Response Tracker - University of Oxford; (vi) Our World in Data – University of Oxford and Global Change Data Lab and (vii) Vaccine Equity Dashboard Data (United Nations Development Programme – UNDP).

GovData360 [10] is a World Bank initiative and presents data on governance-related indicators relating to State capacity, efficiency, accountability, integrity and levels of trust in government.

The Data Futures Platform brings together data from the United Nations (UN) system [11] and from partners to advance integrated development solutions in support of the 2030 Agenda.

United Nations E-Government Knowledgebase [12] is a database that provides public data regarding the governments’ capacity to develop digital solutions for access to information and citizen participation.

The Economist [13] presents data on excess deaths during the pandemic in 223 countries, based on artificial intelligence.

The Oxford COVID-19 Government Response Tracker [14] provides data from over 180 countries on government response capacities in the COVID-19 pandemic.

Our World in Data [15] corresponds to a cooperation between scientists from the University of Oxford and a non-profit organization, Global Change Data Lab., which presents data from indicators related to social disparities, health, climate change and also COVID-19 pandemic indicators.

Vaccine Equity Dashboard Data (United Nations Development Programme – UNDP) [16] is conceived by the United Nations, the World Health Organization and the University of Oxford, which provides data on the progress of vaccination in countries.

### Selection of variables

#### Independent variables

##### Social cohesion

Social cohesion variables were selected based on the concept defined by the United Nations in the Data Futures Platform [11]. Variables: (i) Social capital (Score) (World Bank, 2020); (ii) Media corrupt (Score) (World Bank, 2019); (iii) Engaged society (Score) (World Bank, 2019); (iv) Social safety nets (Score) (World Bank, 2020); (v) Gender equality (Score) (World Bank, 2019); and (vi) Unemployment, youth total (% of the total labour force ages 15–24) (United Nations, 2019); (vii) Equal access index (United Nations, 2019); (viii) Equal distribution of resources index (United Nations, 2019); (ix) Public trust in politicians (Score) (World Bank, 2017–2018); (x) COVID-19-related disorder events (Total number) (United Nations, 2020).

### Risk communication

The risk communication variables were selected based on previous studies [2, 17–19] and the WHO guide [20], which guides risk communication in public health emergencies. This guide presents different aspects related to this type of communication, including relationships of public trust, transparency, access to information, leadership, social participation, community engagement and political coordination. A study defined four components of risk communication: news media exposure, information-gathering ability, trust in the government and trust in news media [21].

In addition to the variables (corrupt media, engaged society and public trust in politicians) already selected to analyse social cohesion, other variables were selected: (i) E-Participation Index (United Nations, 2020); (ii) Transparency of government policymaking (Score) (World Bank, 2017); (iii) Right to information (Score) (World Bank, 2019) and (iv) Policy Coordination (Score) (World Bank, 2020).

Risk communication has 9 principles:1) Timeliness, 2) Transparency, 3) Coordination, 4) Accuracy and consistency, 5) Accountability and integrity, 6) Independence from politics, 7) Responsiveness, 8) Equity, 9) Trust and empathy. Concerning principle 3, effective risk communication depends on good coordination between the different levels of government responsible for disseminating information to the public [22].

Furthermore, the government must create strategies to engage the public and understand their information needs [23].

The demographic, environmental, health, economic and ethnic variables were selected from previously published studies [9, 24, 25]. The variables considered were:

- % population aged > = 65 [26].
- % population living in areas where elevation is < 5 m [26].
- Population density (people per sq. km of land area) [26].
- GDP per capita (current US$) [26].
- % population living in areas where elevation is < 500 m [27].
- Ambient particulate matter pollution (micrograms per cubic meter) [28].
- Age-standardized smoking prevalence (> 15 years) [28].
- High body-mass index [28].
- Asthma prevalence [28].
- Total cancer prevalence [28].
- Chronic obstructive pulmonary disease (COPD) prevalence [28].
- Diabetes mellitus prevalence [28].
- Cardiovascular diseases prevalence [28].
- Tuberculosis prevalence [28].
- Alzheimer’s disease and other dementias prevalence [28].
- Ethnic fractionalization [29].
- People fully vaccinated.
- Delivered population.
- Vaccination policy.
- Administration of the first dose in the country.
- The total number of vaccination doses administered per hundred people at the country level) [30–33].

Factors related to the increased likelihood of patients progressing to the severe form of COVID-19 have been evaluated as possible factors that influence excess mortality due to COVID-19 [34].

Also, the correlation between social cohesion, risk communication variables and vaccination variables was evaluated.

The full list of the variables used is presented in the supplementary material 2.

### Dependent variables

#### Adjusted cumulative infection rate per thousand people and adjusted infection-fatality ratio per thousand infections

This study evaluated two dependent variables related to infection: (i) Adjusted Cumulative infection rate per thousand people and (ii) Adjusted infection-fatality ratio per thousand infections. These variables were assessed for January 1, 2020, until September 30, 2021. One study evaluated the association between cumulative infection rates and infection-fatality ratio due to COVID-19 in 2021 and governance, health, and economic indicators [9]. In this study, accumulated infection rates were adjusted, taking into account possible confounding factors. Therefore, these adjusted rates were used in the present study.

### Daily and cumulative excess deaths per one hundred thousand inhabitants

Excess mortality estimates how many people died during the COVID-19 pandemic, relative to the expected number of deaths, regardless of cause, under normal conditions [3]. Excess mortality corresponds to a more robust assessment of the effects of the pandemic on mortality compared to the number of deaths caused by COVID-19, as it also considers misdiagnosed or misreported deaths and mortality resulting from overburdened health services or exacerbated poverty [13, 35]. For this variable, three periods were used: (i) January 1 to December 7, 2020; (ii) January 1, 2020 to December 6, 2021 and (iii) January 1, 2020 to December 5, 2022. Data from 2017 to 2019 were selected, as they corresponded to the most recent ones, for some indicators. The choice of 2020 is justified by the declaration of a public health emergency by the World Health Organization. Considering the launch and use of the COVID-19 vaccine in 2021 and 2022, these years were included.

### Statistical analysis

Statistical analysis consisted of adjusting a multiple linear regression model for the dependent variables: (i) daily estimated cumulative excess deaths per one hundred thousand inhabitants, in 2020–2022; (ii) adjusted infections per thousand people and (iii) the adjusted infection-fatality ratio per thousand infections), associated with the independent variables: sociodemographic, vaccination, health, social cohesion and risk communication.

The analysis occurred in two stages i.e., (i) simple and (ii) multiple. The simple linear regression was used to select variables to be included in the multiple linear regression model. Initially, simple linear regression models were adjusted for each independent variable. Those in which the *p-value* < 0.05 were included in the multiple linear regression analysis. In the second stage, these variables were adjusted using a multiple linear regression model with a stepwise procedure. Only those variables with *p* < 0.05 remained in the final model. Pearson correlation and biserial correlation coefficients of the variables (social cohesion and communication) and vaccination variables were calculated. In the case of the vaccination policy variable, it was dichotomised as 0 if the vaccination policies were from the year 2021, and 1 if the vaccination policies were from January to June 2022. The variable administration of the first dose in the country was dichotomised into two values: zero if the administration of the first dose occurred from July to December 2020; and one if the administration took place from January to October 2021.

Assumptions of normality, homoscedasticity and linearity were verified through the analysis of residual graphs and probabilistic normal and the identification of possible outliers and leverage points. The basis for the linearity of the independent variables in the linear regression model [36] was presented in supplementary material 3.

Multicollinearity between independent variables was evaluated. It was considered a limit for multicollinearity if the tolerance indicator assumed values greater than 0.60.

The results of the simple linear regression model analysis were not presented.

Analyses were conducted using the SAS 9.4 application [37].

## Results

The adjustment of the stepwise multiple regression model showed that the estimated percentage of the population aged 65 and above, high body mass index, and COVID-19-related disorder events correlate significantly and positively with the daily estimated cumulative excess deaths per one hundred thousand inhabitants in 2020 (*p < 0.0001*, *p < 0.0001 andp = 0.0018*, respectively). On the other hand, the estimated population living in areas where elevation is < 500 m, social safety nets in 2020 and unemployment in 2019 correlate negatively and in a significant way with the daily estimated cumulative excess deaths per one hundred thousand inhabitants in 2020 (*p = 0.0036,p = 0.0047 andp = 0.0449*, respectively) (Table 1). The adjusted model provided an R^2^ = 0.4915.

**Table 1:** Multiple linear regression models for daily and cumulative excess deaths due to COVID-19 per one hundred thousand inhabitants in 2020 by sociodemographic data, vaccination status, health, social cohesion and risk communication variables (*N = 81*)

	Multiple Linear Regression			
Variable	Parameter Estimate	Standard Error	95% CI	*p*-value
Intercept	23,26	18,89	-14,37; 60,90	0,2219
% of population aged > = 65	7,54	1,47	4,62; 10,47	< 0,0001
% population living in areas < 500 m elevation	-0,51	0,17	-0,84; -0,17	0,0036
High body mass index	3,51	0,67	2,17; 4,84	< 0,0001
Social safety nets 2020	-14,26	4,90	-24,02; -4,51	0,0047
Unemployment 2019	-1,18	0,58	-2,34; -0,03	0,0449
COVID related disorder events	0,11	0,03	0,04; 0,18	0,0018

The adjustment of the stepwise multiple regression model showed that the variables high body mass index, cardiovascular disease prevalence and transparency government 2017 correlated significantly and positively with daily excess deaths per one hundred thousand inhabitants in 2021 (*p = 0.0338, p < 0.0001 and p = 0.0417*, respectively). On the other hand, the estimated percentage of the population living in areas where elevation is < 5 m, social capital 2020 and public trust in politicians in 2017 correlated negatively and in a significant way with the daily estimated cumulative excess deaths per one hundred thousand inhabitants in 2021 (*p = 0.0456,p* = 0.0064 and *p = 0.0008*, respectively) (Table 2). The adjusted model provided an R^2^ = 0.6407.

**Table 2:** Multiple linear regression models for daily and cumulative excess deaths due to COVID-19 per one hundred thousand inhabitants in 2021 by sociodemographic data, vaccination status, health, social cohesion and risk communication variables (*N = 87*)

	Multiple Linear Regression				
Variable	Parameter Estimate	Standard Error	95% CI	*p*-value	
Intercept	17,03	79,74	-141,66; 175,72	0,8315	
% population living in areas < 5 m elevation	-3,94	1,97	-8,59; -0,71	0,0456	
High body mass index	2,73	1,26	0,21; 5,24	0,0338	
Cardiovascular diseases prevalence	0,04	0,00	0,03; 0,05	< 0,0001	
Social capital 2020	-29,03	10,36	-49,66; -8,41	0,0064	
Public trust in politicians 2017	-61,62	17,73	-96,91; -26,33	0,0008	
Transparency government 2017	52,97	26,49	0,39; 106,34	0,0417	

The adjustment of the stepwise multiple regression model showed that the percentage of the population aged 65 and above and high body mass correlated significantly and positively with daily excess deaths per one hundred thousand inhabitants in 2022 (*p < 0.0001 andp = 0.0004*, respectively) (Table 3). The adjusted model provided an R^2^ = 0.5982.

**Table 3:** Multiple linear regression models for daily and cumulative excess deaths due to COVID-19 per one hundred thousand inhabitants in 2022 by sociodemographic data, vaccination status, health, social cohesion and risk communication variables (*N = 92*)

	Multiple Linear Regression			
Variable	Parameter Estimate	Standard Error	95% CI	*p*-value
Intercept	70,34	36,16	-7,48; 148,17	0,0759
% of population aged > = 65	27,36	2,90	21,59; 33,13	< 0,0001
High body mass index	5,69	1,56	2,59; 8,78	0,0004
Equal distribution resources index 2019	-274,40	69,22	-411,97; -136,83	0,0001

The adjustment of the stepwise multiple regression model showed that the variables age-standardised, smoking prevalence, high body mass and COVID-19-related disorder events correlated significantly and positively with the adjusted infections per thousand people (*p = 0.0076,p* < 0.0001 and *p = 0.0028*, respectively), with an *N* = 107. On the other hand, total cancer prevalence correlated significantly and negatively with the adjusted infections per thousand people (*p = 0.0350*). As for the social cohesion and risk communication variables, it was positively associated with the variable COVID-19-related disorder events (*p = 0.0028*). The adjusted model provided an R^2^ = 0.3761. The adjusted infection-fatality ratio per thousand infections had no statistically significant association with the independent variables.

Most social cohesion and risk communication variables were associated with better vaccination indicators (Table 4), except for social capital and engaged society, which had no statistically significant association. There was only an association between social capital and delivered population (*p = 0.0002*) and social capital and administration first dose (January to October 2021) (*p < 0.0001*). The higher the gender equality, the better the vaccination indicators, such as people fully vaccinated and the total number of vaccination doses. However, gender equality was associated with the worst indicators of vaccination policies (January to June 2022) and administration first dose (January to October 2021).

**Table 4:** Pearson Correlation and Biserial Correlation coefficients of variables (social cohesion and communication) and vaccination variables, with *p* < 0.0001

Social cohesion and risk communication variable	Vaccination variable	*N*	Correlation Estimate	95% Confidence Limits		*p*-value
Media corrupt 2019	Total number vaccination doses	157	0.32299	0.175212	0.456528	< 0.0001
Social safety nets 2020	Total number vaccination doses	132	0.55526	0.424684	0.663210	< 0.0001
Gender equality 2019	Total number vaccination doses	157	0.47294	0.341612	0.586158	< 0.0001
Equal distribution resources index	Total number vaccination doses	171	0.54258	0.427300	0.640498	< 0.0001
Public trust in politicians 2017	Total number vaccination doses	136	0.41229	0.262155	0.542945	< 0.0001
E participation index 2020	Total number vaccination doses	188	0.58953	0.487557	0.675640	< 0.0001
Transparency government 2017	Total number vaccination doses	136	0.53549	0.403537	0.645629	< 0.0001
Right information 2019	Total number vaccination doses	125	0.52298	0.382503	0.639828	< 0.0001
Policy coordination 2019	Total number vaccination doses	135	0.41677	0.266585	0.547196	< 0.0001
Social safety nets 2020	People fully vaccinated	132	0.54761	0.415624	0.657003	< 0.0001
Gender equality 2019	People fully vaccinated	157	0.44857	0.313998	0.565479	< 0.0001
Equal distribution resources index	People fully vaccinated	171	0.52031	0.401596	0.621828	< 0.0001
Public trust in politicians 2017	People fully vaccinated	136	0.37450	0.220035	0.510639	< 0.0001
E participation index 2020	People fully vaccinated	188	0.57575	0.471493	0.664141	< 0.0001
Transparency government 2017	People fully vaccinated	136	0.49195	0.352830	0.609782	< 0.0001
Right information 2019	People fully vaccinated	125	0.48095	0.333510	0.605424	< 0.0001
Policy coordination 2019	People fully vaccinated	135	0.41464	0.264188	0.545387	< 0.0001
Media corrupt 2019	Delivered population	156	0.43203	0.294915	0.551719	< 0.0001
Social safety nets 2020	Delivered population	131	0.65451	0.544067	0.742663	< 0.0001
Gender equality 2019	Delivered population	156	0.56403	0.446455	0.662452	< 0.0001
Equal access index 2019	Delivered population	170	0.34467	0.204776	0.470760	< 0.0001
Equal distribution resources index	Delivered population	170	0.57857	0.468889	0.670680	< 0.0001
E participation index 2020	Delivered population	188	0.56731	0.461687	0.657077	< 0.0001
Transparency government 2017	Delivered population	135	0.44529	0.298815	0.571269	< 0.0001
Right information 2019	Delivered population	125	0.52644	0.386564	0.642634	< 0.0001
Policy coordination 2019	Delivered population	133	0.50985	0.371895	0.625763	< 0.0001
Gender equality 2019	Vaccination policies(Jan to Jun 2022)	147	-0.18035	-0.332537	-0.019015	0.0281
E participation index 2020	Vaccination policies(Jan to Jun 2022)	167	-0.30802	-0.439329	-0.163814	< 0.0001
Social capital 2020	Administration first dose(Jan to Out 2021)	128	-0.35121	-0.494595	-0.189208	< 0.0001
Media corrupt 2019	Administration first dose(Jan to Out 2021)	151	-0.41647	-0.540256	-0.275034	< 0.0001
Social safety nets 2020	Administration first dose(Jan to Out 2021)	126	-0.52335	-0.639700	-0.383552	< 0.0001
Gender equality 2019	Administration first dose(Jan to Out 2021)	151	-0.50662	-0.616466	-0.377438	< 0.0001
Equal access index 2019	Administration first dose(Jan to Out 2021)	165	-0.38944	-0.511771	-0.251626	< 0.0001
Equal distribution resources ind	Administration first dose(Jan to Out 2021)	165	-0.52609	-0.628369	-0.405938	< 0.0001
E participation index 2020	Administration first dose(Jan to Out 2021)	185	-0.54587	-0.639753	-0.435928	< 0.0001
Right information 2019	Administration first dose(Jan to Out 2021 )	121	-0.54577	-0.659971	-0.406911	< 0.0001

## Discussion

One of the key findings of the present study was that the greater the number of disorder events related to COVID-19, the greater the excess mortality, in 2020, and the adjusted cumulative infection rate per thousand people due to COVID-19. It may be that this fact became more evident in 2020 because at the beginning of the pandemic, the virus and the disease were not known and people saw their jobs and lives threatened, due to government measures, which influenced waves of protests. A study revealed that in India, Israel and Mexico, where the largest number of protests in the world occurred during the COVID-19 pandemic, restrictive government measures to contain the spread of the COVID-19 virus, such as quarantine, may have influenced people to organise themselves to protest against these measures, because they felt entitled to go out to work to maintain basic conditions, such as to purchase food products. Thus, people have organised themselves to protest. In the crowds at these events, the infection rate may have increased. These events can impact adherence to government measures and, consequently, increase the number of cases or deaths due to COVID-19, in agreement with the results presented by the present research. Strengthening trust between government and citizens can improve citizens’ perception regarding the brevity of measures and the importance of achieving the good of all [38].

The COVID-19-related disorder events may also be related to the country’s economic situation, aggravated by the pandemic. The results of the present research showed that there was a negative association between the unemployment rate and excess mortality due to COVID-19. This was not an expected result, as in an unstable economic scenario, one could think that unemployment could force families to expose themselves to more risks, such as going out to look for a job or continuing in the informal market. This can reduce adherence to recommendations. According to a study, unemployment is a factor that impacted the efficiency of countries in the face of the difficulties imposed by the pandemic [39]. Alternatively, it is possible that providing economic support in countries with limited resources and high unemployment rates may have boosted public trust in the government. Initially, it may have given the impression that the government was prioritising the most vulnerable members of society. This confidence can affect acceptance and adherence to governments’ measures to control the pandemic.

Social safety nets are negatively proportional and associated with excess mortality due to COVID-19, according to the results of the present study. This was an expected result, as these safety nets are policy-related, for example, cash or food transfer programs or education-related initiatives, to reduce social risks such as unemployment and poverty. A study conducted in Malawi showed that individuals benefiting from social safety nets were less likely to reduce food consumption and need to use up investments (savings) during the COVID-19 pandemic [40]. Another study conducted in nine African countries, showed an association, albeit weak, between social safety nets and reduced food deprivation [41]. In the concept of social safety nets, defined by the World Bank, the highest score corresponds to the compensation of social risks through programs or policies, especially concerning health care. Healthcare safety nets were defined by one study as the provision of health support for those with difficulties in accessing health services. According to the definition proposed by this study, which was a systematic literature review, healthcare safety nets can result in decreased morbidity and mortality [42]. Social safety nets can be strategies for coping with a pandemic, as they can reduce social and economic vulnerability, which can impact following governmental measures and, consequently, better controlling a pandemic.

The results of this study showed that the higher the level of interpersonal trust in the government, the lower the excess mortality due to COVID-19. They agree with the findings from previous studies about the level of trust in the government. However, the dimensions of social capital influenced mortality due to COVID-19 differently. The literature showed that trust in government and politicians was associated with better adherence to government measures to control the pandemic [43–45] and reduced mortality rates due to COVID-19 [46–48]. On the other hand, a high level of trust in the government can make it difficult to enforce strict measures. For example, it may be that countries with a higher level of trust in government are those with strong liberal tendencies, which can make it difficult to enforce these measures, as individuals may find their freedoms threatened. The implementation of very strict measures can influence a population’s level of confidence in the government and reduce adherence to these measures [46].

Social capital is related to the level of trust between people and the need for mutual help in a crisis. This can impact compliance, such as wearing a mask in public, as it is related to concern for the other and the good of all. One study showed that in Japan, different dimensions of social capital had different effects on mortality due to COVID-19. The level of reciprocity and trust in the government were associated with a lower mortality rate due to COVID-19, from October to December 2020 and from January to March 2021. The other evaluated dimensions such as trust in neighbours, neighbourhood ties and social participation, were not associated with COVID-19 deaths [49]. On the other hand, another study indicated that societies with a greater capacity to organise themselves into groups had more deaths due to COVID-19. One explanation is that the identification and proximity of the associated groups may compromise physical distancing. Participation in these groups, makes people feel welcomed and protected [50]. Therefore, social capital influences the behaviour of people during a pandemic in different ways.

Although the results of the present study did not indicate an association between the adjusted cumulative infection rate per thousand people due to COVID-19 and social capital, a study, which assessed data from 177 countries, showed that trust in the government and the people around us is correlated with lower infection rates due to COVID-19. The results suggest that, if countries achieved trust in government as can be seen in Denmark, there would be 13% fewer infections on the planet. Regarding the ability to trust the other, based on Denmark’s level, there would be 40% fewer infections [51].

The present study showed that the greater the transparency of the government, the greater the excess mortality due to COVID-19. A transparent government tends to disclose data, such as the number of cases and deaths, regardless of whether this can compromise the government’s image concerning its performance and efficiency in controlling the pandemic. In contrast, a study showed a different result, which indicated that the lack of information or disclosure of unreliable information was associated with a greater number of cases of respiratory infections, including COVID-19. However, this same study showed no association between mortality related to these infections and the transparency of information [52]. This may be due to the difference between transparency and reliability, as a government can be transparent, but not use strategies to convey information in a way that is clear or that reaches the target population. Furthermore, transparency does not mean the dissemination of reliable information.

Choosing a strategy for communicating reliable information transparently, including through social media, strengthens people’s trust in government. Also, the creation of mechanisms for the active participation of individuals in the evaluation of government actions improves confidence and can help countries increase their capacity to prepare for future pandemics [17, 44, 53].

Despite the results of the present study showing that there was no significant association between the equal access index and excess mortality due to COVID-19, a study suggested that equity of access to health care implies a 0.38% reduction in the mortality rate due to COVID-19 when considering an increase in the standard deviation greater than the average value of this index. Still, this study indicates that social aspects such as trust in government and social capital are less important than the infrastructure of health systems and access to services, as countries that are largely egalitarian, where higher levels of trust are suggested, had greater difficulty in balancing different interests, such as reducing food insecurity, implementing more restrictive measures and making measures more flexible to guarantee individual rights [54].

The definition of the equal access index from the United Nations, which measures how power is distributed to needy and minority populations, may not have a positive relationship to access to health care. Furthermore, one does not know the exact measure of this power. Thus, it may have impacted the study results. A higher index of equal distribution of resources in a country, on the other hand, had a negatively proportional association with excess mortality due to COVID-19, according to the results of this study. The definition of this index corresponds to the measure of perception of how well resources are distributed in a country. This measure may have a closer relationship with the resources used in the pandemic, such as those related to health infrastructure (number of hospital beds, equipment, health professionals, etc.).

The variables of social capital and engaged society were not associated with most vaccination variables, including the total number of doses and fully vaccinated people. One study found that the greater the trust in government, the greater the vaccination. However, it was negatively proportional when the association between trust in others and vaccination was analysed. One hypothesis is that other factors influence this relationship, such as religion [55]. Another study, which was conducted in the United States, suggested that social capital has strongly contributed to the inequalities of vaccination uptake in the USA. Furthermore, this study reinforces that other factors influence this relationship, such as level of education and race [56]. By not identifying the association between the various sources, this present study cannot be controlled by factors that influence this relationship. The relationship between the population and the government may be less influenced by other factors.

Most social cohesion and risk communication variables were associated with better vaccination indicators, including the people fully vaccinated. Not only do social cohesion and risk communication seem to influence vaccination indicators, but also countries with better governance indicators had a higher number of people fully vaccinated [57].

According to the results of the present study, variables related to risk communication, such as corrupt media, right to information and social participation index, were not associated with excess mortality and adjusted cumulative infection rate per thousand people. However, government transparency was related to excess mortality. Other factors related to communication may not have been analyzed in the study. From this, more research on the role of risk communication in pandemics must be carried out.

One published study left an important recommendation, that risk communication is a process of exchange between government and the citizen, and that citizen engagement in this process, their assessment and perceptions are vital [58].

The present study has some limitations. The ecologic design of the study implies that the evidence may suffer from ecological bias. Thus, it was not possible to infer causal relationships and the correlation coefficient analysis does not imply a cause-and-effect analysis. Other variables, which were not considered in the analysis, may have influenced the results of the study. For example, the COVID-19 infection rate is not comparable over time or between states, as it was influenced by the testing strategy or testing capacities. The reliability of the data sources raises some concerns. The data may be influenced by government officials. The study results may have been impacted by missing data and temporal variation in COVID-19 outcomes. Risk perception is a factor that influences the risk communication process and the behaviour of individuals when faced with threats [21]. Therefore, these factors do not pretend to represent the whole complexity of the topic. It has to be emphasized that our analysis did not take into account relevant socio-economic indicators and cultural [59]. The results may have been affected by missing data and temporal variation in COVID-19 outcomes.

## Conclusions

Social cohesion and risk communication, in their dimensions (public trust in politicians, trust in others, social safety nets, and equal distribution of resources index), were associated with lower excess mortality due to COVID-19. The number of COVID-19-related disorder events and government transparency, on the other hand, were associated with higher excess mortality due to COVID-19. Regarding unemployment, the lower the percentage of people, the higher the excess mortality due to COVID-19. Most of the social cohesion and risk communication variables were associated with better immunization indicators, except for social capital and engaged society, which had no statistically significant association. Public trust in politicians and others, equal distribution of resources and a government that cares about the most vulnerable starting with the implementation of programs, such as cash transfers and combating food insecurity, were factors that reduced the excess mortality due to COVID-19. Future studies are important to investigate other social cohesion and risk communication variables that may have contributed to effective responses in the COVID-19 pandemic. The instruments for assessing countries’ preparedness and response capacity of countries facing public health emergencies should be reviewed, and these factors of social cohesion and risk communication should be considered as potential predictors of preparedness and response. Trust and government transparency were crucial factors in tackling the pandemic, but due to the complexity of the topic, the results cannot be generalised. Future studies can investigate other factors, which may have impacted Covid-19 outcomes, such as social, economic, and cultural differences. Countries, especially those with limited resources and marked by social, economic, and health inequalities, should invest in strengthening social cohesion and risk communication, which are strategies to better cope with future pandemics.

The complete list of 213 countries is presented in supplementary material 1.

The definitions, database and year of the variables are presented in supplementary material 2.

The basis for the linearity of the independent variables in the linear regression model is presented in supplementary material 3.

### Electronic supplementary material

Below is the link to the electronic supplementary material.

Supplementary Material 1

Supplementary Material 2

Supplementary Material 3

## Data Availability

The datasets used and/or analysed during the current study available from the corresponding author on reasonable request. All data are available in publicly accessible databases, which are reported in the methods section.
