# Supplementary material for: The impact of social cohesion and risk communication on excess mortality due to COVID-19 in 213 countries: a retrospective analysis
Source: BMC Public Health. 2024 Jun 14;24:1598. doi: 10.1186/s12889-024-19076-7 (PMC11179214; doi:10.1186/s12889-024-19076-7)
Supplement: Supplementary file 1 — Supplementary Material 1 [file 12889_2024_19076_MOESM1_ESM.docx]

| **Country**  **Supplementary material 1: Complete list of countries, by region.** | **Region** |
| --- | --- |
| Afghanistan | SOUTH ASIA |
| Albania | EUROPE AND CENTRAL ASIA |
| Algeria | MIDDLE EAST AND NORTH AFRICA |
| American Samoa | EAST ASIA AND PACIFIC |
| Andorra | EUROPE AND CENTRAL ASIA |
| Angola | SUB-SAHARAN AFRICA |
| Anguilla | EUROPE AND CENTRAL ASIA |
| Antigua and Barbuda | LATIN AMERICA AND THE CARIBBEAN |
| Argentina | LATIN AMERICA AND THE CARIBBEAN |
| Armenia | EUROPE AND CENTRAL ASIA |
| Aruba | LATIN AMERICA AND THE CARIBBEAN |
| Australia | EAST ASIA AND PACIFIC |
| Austria | EUROPE AND CENTRAL ASIA |
| Azerbaijan | EUROPE AND CENTRAL ASIA |
| Bahamas, The | LATIN AMERICA AND THE CARIBBEAN |
| Bahrain | MIDDLE EAST AND NORTH AFRICA |
| Bangladesh | SOUTH ASIA |
| Barbados | LATIN AMERICA AND THE CARIBBEAN |
| Belarus | EUROPE AND CENTRAL ASIA |
| Belgium | EUROPE AND CENTRAL ASIA |
| Belize | LATIN AMERICA AND THE CARIBBEAN |
| Benin | SUB-SAHARAN AFRICA |
| Bermuda | NORTH AMERICA |
| Bhutan | SOUTH ASIA |
| Bolivia | LATIN AMERICA AND THE CARIBBEAN |
| Bosnia and Herzegovina | EUROPE AND CENTRAL ASIA |
| Botswana | SUB-SAHARAN AFRICA |
| Brazil | LATIN AMERICA AND THE CARIBBEAN |
| Brunei Darussalam | EAST ASIA AND PACIFIC |
| Bulgaria | EUROPE AND CENTRAL ASIA |
| Burkina Faso | SUB-SAHARAN AFRICA |
| Burundi | SUB-SAHARAN AFRICA |
| Cabo Verde | SUB-SAHARAN AFRICA |
| Cambodia | EAST ASIA AND PACIFIC |
| Cameroon | SUB-SAHARAN AFRICA |
| Canada | NORTH AMERICA |
| Cayman Islands | LATIN AMERICA AND THE CARIBBEAN |
| Central African Republic | SUB-SAHARAN AFRICA |
| Chad | SUB-SAHARAN AFRICA |
| Chile | LATIN AMERICA AND THE CARIBBEAN |
| China | EAST ASIA AND PACIFIC |
| Colombia | LATIN AMERICA AND THE CARIBBEAN |
| Comoros | SUB-SAHARAN AFRICA |
| Congo, Dem. Rep. | SUB-SAHARAN AFRICA |
| Congo, Rep. | SUB-SAHARAN AFRICA |
| Cook Islands | EAST ASIA AND PACIFIC |
| Costa Rica | LATIN AMERICA AND THE CARIBBEAN |
| Cote d'Ivoire | SUB-SAHARAN AFRICA |
| Croatia | EUROPE AND CENTRAL ASIA |
| Cuba | LATIN AMERICA AND THE CARIBBEAN |
| Cyprus | EUROPE AND CENTRAL ASIA |
| Czech Republic | EUROPE AND CENTRAL ASIA |
| Denmark | EUROPE AND CENTRAL ASIA |
| Djibouti | MIDDLE EAST AND NORTH AFRICA |
| Dominica | LATIN AMERICA AND THE CARIBBEAN |
| Dominican Republic | LATIN AMERICA AND THE CARIBBEAN |
| Ecuador | LATIN AMERICA AND THE CARIBBEAN |
| Egypt, Arab Rep. | MIDDLE EAST AND NORTH AFRICA |
| El Salvador | LATIN AMERICA AND THE CARIBBEAN |
| Equatorial Guinea | SUB-SAHARAN AFRICA |
| Eritrea | SUB-SAHARAN AFRICA |
| Estonia | EUROPE AND CENTRAL ASIA |
| Eswatini | SUB-SAHARAN AFRICA |
| Ethiopia | SUB-SAHARAN AFRICA |
| Fiji | EAST ASIA AND PACIFIC |
| Finland | EUROPE AND CENTRAL ASIA |
| France | EUROPE AND CENTRAL ASIA |
| French Guiana | EUROPE AND CENTRAL ASIA |
| Gabon | SUB-SAHARAN AFRICA |
| Gambia, The | SUB-SAHARAN AFRICA |
| Georgia | EUROPE AND CENTRAL ASIA |
| Germany | EUROPE AND CENTRAL ASIA |
| Ghana | SUB-SAHARAN AFRICA |
| Greece | EUROPE AND CENTRAL ASIA |
| Greenland | EUROPE AND CENTRAL ASIA |
| Grenada | LATIN AMERICA AND THE CARIBBEAN |
| Guam | EAST ASIA AND PACIFIC |
| Guatemala | LATIN AMERICA AND THE CARIBBEAN |
| Guinea | SUB-SAHARAN AFRICA |
| Guinea-Bissau | SUB-SAHARAN AFRICA |
| Guyana | LATIN AMERICA AND THE CARIBBEAN |
| Haiti | LATIN AMERICA AND THE CARIBBEAN |
| Honduras | LATIN AMERICA AND THE CARIBBEAN |
| Hong Kong SAR, China | EAST ASIA AND PACIFIC |
| Hungary | EUROPE AND CENTRAL ASIA |
| Iceland | EUROPE AND CENTRAL ASIA |
| India | SOUTH ASIA |
| Indonesia | EAST ASIA AND PACIFIC |
| Iran, Islamic Rep. | MIDDLE EAST AND NORTH AFRICA |
| Iraq | MIDDLE EAST AND NORTH AFRICA |
| Ireland | EUROPE AND CENTRAL ASIA |
| Israel | MIDDLE EAST AND NORTH AFRICA |
| Italy | EUROPE AND CENTRAL ASIA |
| Jamaica | LATIN AMERICA AND THE CARIBBEAN |
| Japan | EAST ASIA AND PACIFIC |
| Jersey, Channel Islands | EUROPE AND CENTRAL ASIA |
| Jordan | MIDDLE EAST AND NORTH AFRICA |
| Kazakhstan | EUROPE AND CENTRAL ASIA |
| Kenya | SUB-SAHARAN AFRICA |
| Kiribati | EAST ASIA AND PACIFIC |
| Korea, Dem. People's Rep. | EAST ASIA AND PACIFIC |
| Korea, Rep. | EAST ASIA AND PACIFIC |
| Kosovo | EUROPE AND CENTRAL ASIA |
| Kuwait | MIDDLE EAST AND NORTH AFRICA |
| Kyrgyz Republic | EUROPE AND CENTRAL ASIA |
| Lao PDR | EAST ASIA AND PACIFIC |
| Latvia | EUROPE AND CENTRAL ASIA |
| Lebanon | MIDDLE EAST AND NORTH AFRICA |
| Lesotho | SUB-SAHARAN AFRICA |
| Liberia | SUB-SAHARAN AFRICA |
| Libya | MIDDLE EAST AND NORTH AFRICA |
| Liechtenstein | EUROPE AND CENTRAL ASIA |
| Lithuania | EUROPE AND CENTRAL ASIA |
| Luxembourg | EUROPE AND CENTRAL ASIA |
| Macao SAR, China | EAST ASIA AND PACIFIC |
| Madagascar | SUB-SAHARAN AFRICA |
| Malawi | SUB-SAHARAN AFRICA |
| Malaysia | EAST ASIA AND PACIFIC |
| Maldives | SOUTH ASIA |
| Mali | SUB-SAHARAN AFRICA |
| Malta | MIDDLE EAST AND NORTH AFRICA |
| Marshall Islands | EAST ASIA AND PACIFIC |
| Martinique | EUROPE AND CENTRAL ASIA |
| Mauritania | SUB-SAHARAN AFRICA |
| Mauritius | SUB-SAHARAN AFRICA |
| Mexico | LATIN AMERICA AND THE CARIBBEAN |
| Micronesia, Fed. Sts. | EAST ASIA AND PACIFIC |
| Moldova | EUROPE AND CENTRAL ASIA |
| Monaco | EUROPE AND CENTRAL ASIA |
| Mongolia | EAST ASIA AND PACIFIC |
| Montenegro | EUROPE AND CENTRAL ASIA |
| Morocco | MIDDLE EAST AND NORTH AFRICA |
| Mozambique | SUB-SAHARAN AFRICA |
| Myanmar | EAST ASIA AND PACIFIC |
| Namibia | SUB-SAHARAN AFRICA |
| Nauru | EAST ASIA AND PACIFIC |
| Nepal | SOUTH ASIA |
| Netherlands | EUROPE AND CENTRAL ASIA |
| New Zealand | EAST ASIA AND PACIFIC |
| Nicaragua | LATIN AMERICA AND THE CARIBBEAN |
| Niger | SUB-SAHARAN AFRICA |
| Nigeria | SUB-SAHARAN AFRICA |
| Niue | EAST ASIA AND PACIFIC |
| North Macedonia | EUROPE AND CENTRAL ASIA |
| Norway | EUROPE AND CENTRAL ASIA |
| Oman | MIDDLE EAST AND NORTH AFRICA |
| Pakistan | SOUTH ASIA |
| Palau | EAST ASIA AND PACIFIC |
| Panama | LATIN AMERICA AND THE CARIBBEAN |
| Papua New Guinea | EAST ASIA AND PACIFIC |
| Paraguay | LATIN AMERICA AND THE CARIBBEAN |
| Peru | LATIN AMERICA AND THE CARIBBEAN |
| Philippines | EAST ASIA AND PACIFIC |
| Poland | EUROPE AND CENTRAL ASIA |
| Portugal | EUROPE AND CENTRAL ASIA |
| Puerto Rico | LATIN AMERICA AND THE CARIBBEAN |
| Qatar | MIDDLE EAST AND NORTH AFRICA |
| Reunion | EUROPE AND CENTRAL ASIA |
| Romania | EUROPE AND CENTRAL ASIA |
| Russian Federation | EUROPE AND CENTRAL ASIA |
| Rwanda | SUB-SAHARAN AFRICA |
| Samoa | EAST ASIA AND PACIFIC |
| San Marino | EUROPE AND CENTRAL ASIA |
| Sao Tome and Principe | SUB-SAHARAN AFRICA |
| Saudi Arabia | MIDDLE EAST AND NORTH AFRICA |
| Senegal | SUB-SAHARAN AFRICA |
| Serbia | EUROPE AND CENTRAL ASIA |
| Seychelles | SUB-SAHARAN AFRICA |
| Sierra Leone | SUB-SAHARAN AFRICA |
| Singapore | EAST ASIA AND PACIFIC |
| Slovak Republic | EUROPE AND CENTRAL ASIA |
| Slovenia | EUROPE AND CENTRAL ASIA |
| Solomon Islands | EAST ASIA AND PACIFIC |
| Somalia | SUB-SAHARAN AFRICA |
| South Africa | SUB-SAHARAN AFRICA |
| South Sudan | SUB-SAHARAN AFRICA |
| Spain | EUROPE AND CENTRAL ASIA |
| Sri Lanka | SOUTH ASIA |
| St. Kitts and Nevis | LATIN AMERICA AND THE CARIBBEAN |
| St. Lucia | LATIN AMERICA AND THE CARIBBEAN |
| St. Vincent and the Grenadines | LATIN AMERICA AND THE CARIBBEAN |
| Sudan | SUB-SAHARAN AFRICA |
| Suriname | LATIN AMERICA AND THE CARIBBEAN |
| Sweden | EUROPE AND CENTRAL ASIA |
| Switzerland | EUROPE AND CENTRAL ASIA |
| Syrian Arab Republic | MIDDLE EAST AND NORTH AFRICA |
| Taiwan, China | EAST ASIA AND PACIFIC |
| Tajikistan | EUROPE AND CENTRAL ASIA |
| Tanzania | SUB-SAHARAN AFRICA |
| Thailand | EAST ASIA AND PACIFIC |
| Timor-Leste | EAST ASIA AND PACIFIC |
| Togo | SUB-SAHARAN AFRICA |
| Tonga | EAST ASIA AND PACIFIC |
| Trinidad and Tobago | LATIN AMERICA AND THE CARIBBEAN |
| Tunisia | MIDDLE EAST AND NORTH AFRICA |
| Turkiye | EUROPE AND CENTRAL ASIA |
| Turkmenistan | EUROPE AND CENTRAL ASIA |
| Tuvalu | EAST ASIA AND PACIFIC |
| Uganda | SUB-SAHARAN AFRICA |
| Ukraine | EUROPE AND CENTRAL ASIA |
| United Arab Emirates | MIDDLE EAST AND NORTH AFRICA |
| United Kingdom | EUROPE AND CENTRAL ASIA |
| United States | NORTH AMERICA |
| Uruguay | LATIN AMERICA AND THE CARIBBEAN |
| Uzbekistan | EUROPE AND CENTRAL ASIA |
| Vanuatu | EAST ASIA AND PACIFIC |
| Venezuela, RB | LATIN AMERICA AND THE CARIBBEAN |
| Vietnam | EAST ASIA AND PACIFIC |
| Virgin Islands (U.S.) | LATIN AMERICA AND THE CARIBBEAN |
| West Bank and Gaza | MIDDLE EAST AND NORTH AFRICA |
| Yemen, Rep. | MIDDLE EAST AND NORTH AFRICA |
| Zambia | SUB-SAHARAN AFRICA |
| Zimbabwe | SUB-SAHARAN AFRICA |
