# Supplementary material for: The impact of social cohesion and risk communication on excess mortality due to COVID-19 in 213 countries: a retrospective analysis
Source: BMC Public Health. 2024 Jun 14;24:1598. doi: 10.1186/s12889-024-19076-7 (PMC11179214; doi:10.1186/s12889-024-19076-7)
Supplement: Supplementary file 2 — Supplementary Material 2 [file 12889_2024_19076_MOESM2_ESM.docx]

| **Supplementary material 2: Variables, source, description and year** | | | |
| --- | --- | --- | --- |
| **Variable** | **Source** | **Description** | **Year** |
| Public trust in politicians | World Bank | *“In your country, how do you rate the ethical standards of politicians? [1 = extremely low; 7 = extremely high] \| 2016–17 weighted average* | 2017 |
| Social capital | World Bank | *the level of trust between citizens, which fosters cooperation and mutual support for purposes of self-help, rather than primarily to further political objectives.*  *Social capital may also be based on cultural patterns of interaction characterizing traditional societies. Please indicate · to what extent there is a sense of solidarity and trust among the citizens (as measured by public opinion surveys) · to what extent there is a voluntary and autonomous organization of cultural, environmental or social associations. Scale: 1-There is a very low level of trust among the population, and civic self-organization is rudimentary; 2; 3 (intermediate scores); 4-There is a fairly low level of trust among the population. The small number of autonomous, self-organized groups, associations and organizations is unevenly distributed or spontaneous and temporary; 5; 6 (intermediate scores); 7-There is a fairly high level of trust among the population and a substantial number of autonomous, self-organized groups, associations and organizations; 8; 9 (intermediate scores); 10-There is a very high level of trust among the population and a large number of autonomous, self-organized groups, associations and organizations* | 2020 |
| Right to information | World Bank | *Measures whether requests for information held by a government agency are granted, whether these requests are granted within a reasonable time period, if the information provided is pertinent and complete, and if requests for information are granted at a reasonable cost and without having to pay a bribe. It also measures whether people are aware of their right to information, and whether relevant records are accessible to the public upon request. The scores corresponds to the total.* | 2020 |
| Engaged society | World Bank | *Responses:*  *0: Public deliberation is never, or almost never, allowed.*  *1: Some limited public deliberations are allowed but the public below the elite level is almost always either unaware of major policy debates or unable to*  *take part in them.*  *2: Public deliberation is not repressed but infrequent; and non-elite actors are typically controlled and/or constrained by the elites.*  *3: Public deliberation is actively encouraged and some autonomous non-elite groups participate, but it is confined to a small slice of specialized groups that tend to be the same across issue-areas.*  *4: Public deliberation is actively encouraged and a relatively broad segment of non-elite groups often participate; these vary with different issue-areas.*  *5: Large numbers of non-elite groups as well as ordinary people tend to discuss major policies among themselves, in the media, in associations or neighbourhoods and in the streets. Grassroots deliberation is common and unconstrained.*  *Clarification*  *This question refers to deliberation as manifest in discussion, debate and other public forums such as popular media. The scores range from 0 to 1, where 1 signifies the highest possible score and 0 signifies the lowest possible score.* | 2020 |
| Social safety nets | World Bank | *This question refers to social safety nets which compensate for the social risks of the capitalist economic system, such as unemployment or poverty, and which alleviate handicaps such as old age, illness or disability. In your answer, please make sure to comment on: · the forms of compensation: cash and conditional cash transfers, subsidies, waivers, food programs, educational or empowerment initiatives · the funding: tax, redistribution, pay-as-you-go, investment funds · the structure of the welfare regime: public, private or both · the coverage/inclusiveness of social safety nets (where applicable, please also comment on the access of non-citizens to social safety nets). Note: In countries that do not provide comprehensive, state-funded welfare systems, social safety nets can refer to those arrangements that are functionally equivalent to formal compensation for social risks. Social safety nets may also comprise family, clan or village structures, if these structures can provide viable compensation on a broad scale for risks. Scale: 1-Social safety nets do not exist. Poverty is combated hardly at all, or only ad hoc; 2; 3 (intermediate scores); 4-Social safety nets are rudimentary and cover only few risks for a limited number of beneficiaries. The majority of the population is at risk of poverty; 5; 6 (intermediate scores); 7-Social safety nets are well developed, but do not cover all risks for all strata of the population. A significant part of the population is still at risk of poverty; 8; 9 (intermediate scores); 10-Social safety nets are comprehensive and compensate for social risks, especially nationwide health care and a well-focused prevention of poverty.* | 2020 |
| Policy coordination | World Bank | *As many policies have conflicting objectives, reflect competing political interests and affect other policies, the government has to ensure that its overall policy is coherent. Successful coordination should: · assure that trade-offs between policy goals are well balanced · introduce horizontal forms of coordination to mediate between different departments of the state administration · ascribe responsibilities in a transparent manner to avoid the negligence of tasks, redundancies or friction be- tween different government branches. Various coordination styles— hierarchic-bureaucratic, informal-network, personalist, centralized, decentralized etc. — are possible and may be functionally equivalent. What matters is their impact on policy coherence. Scale: 1-The government fails to coordinate conflicting objectives. Its policies thwart and damage each other. The executive is fragmented into rival fiefdoms that counteract each other; 2; 3 (intermediate scores); 4-The government often fails to coordinate between conflicting objectives. Different parts of the government tend to compete among each other, and some policies have counterproductive effects on other policies; 5; 6 (intermediate scores); 7-The government tries to coordinate conflicting objectives, but friction, redundancies and gaps in task assignment are significant; 8; 9 (intermediate scores); 10-The government coordinates conflicting objectives effectively and acts in a coherent manner* | 2020 |
| Transparency of government policymaking | World Bank | *In your country, how easy is it for companies to obtain information about changes in government policies and regulations affecting their activities? [1 = extremely difficult; 7 = extremely easy] \| 2016–17 weighted average* | 2017 |
| Equal distribution of resources index | United Nations | *How equal is the distribution of resources? This component measures the extent to which resources — both tangible and*  *intangible — are distributed in society. An equal distribution of resources supports*  *egalitarian democracy in two ways. First, lower poverty rates and the distribution of goods*  *and services such as food, water, housing, education and healthcare ensure that all*  *individuals are capable of participating in politics and government. In short, basic needs*  *must be met in order for individuals to effectively exercise their rights and freedoms see, for*  *example, Sen 1999, Maslow 1943. Second, high levels of resource inequality undermine the*  *ability of poorer populations to participate meaningfully Aristotle, Dahl 2006. The scores range from 0 to 1, where 1 signifies the highest possible score and 0 signifies the lowest possible score.* | 2019 |
| Equal access index | United Nations | *How equal is access to power? The Equal Access subcomponent is based on the idea that neither the protections*  *of rights and freedoms nor the equal distribution of resources is sufficient to ensure adequate representation. Ideally, all groups should enjoy equal de facto capabilities to participate, to*  *serve in positions of political power, to put issues on the agenda, and to influence policymaking. The scores range from 0 to 1, where 1 signifies the highest possible score and 0 signifies the lowest possible score.* | 2019 |
| E-Participation Index | United Nations | *The purpose of this measure is not to prescribe any specific practice, but rather to offer insight into how different countries are using online tools in promoting interaction between the government and its people, as well as among the people, for the benefit of all. The scores range from 0 to 1, where 1 signifies the highest possible score and 0 signifies the lowest possible score.* | 2020 |
| Media corrupt | World Bank | *Question: Do journalists, publishers or broadcasters accept payments in exchange for altering news coverage? Responses: 0: The media are so closely directed by the government that any such payments would be either unn... Read More necessary to ensure pro-government coverage or ineffective in producing anti-government coverage. 1: Journalists, publishers and broadcasters routinely alter news coverage in exchange for payments. 2: It is common, but not routine, for journalists, publishers and broadcasters to alter news coverage in exchange for payments. 3: It is not normal for journalists, publishers and broadcasters to alter news coverage in exchange for payments, but it happens occasionally without anyone being punished. 4: Journalists, publishers and broadcasters rarely alter news coverage in exchange for payments and if it becomes known, someone is punished for it. The scores range from 0 to 1, where 1 signifies the highest possible score and 0 signifies the lowest possible score.* | 2019 |
| Gender equality | World Bank | *This criterion assesses the extent to which the country has enacted and put in place institutions and programs to enforce laws and policies that (a) promote equal access for men and women to human capital development; (b) promote equal access for men and women to productive and economic resources; and (c) give men and women equal status and protection under the law.* *The scores range from 1 (low) to 6 (high).* | 2019 |
| Unemployment youth total | United Nations | *% of total labor force ages 15-24* | 2019 |
| Covid-related disorder events | United Nations | *Countries where the number of demonstrations increased since the pandemic declaration* | 2020 |
| ^1^Infection-fatality ratio |  | *The IFRs were calculated by applying a 9-day lag to our daily infections to account for the delay between infection and death, calculating the sum of infections and deaths, and then dividing the cumulative deaths over the cumulative lagged infections. For this research, Adjusted IFR per 1000 infections will be used. IFR has been adjusted to population density, gross domestic product (GDP), altitude, and seasonality— factors that might increase transmission—and age, age-standardised chronic obstructive pulmonary disease (COPD) prevalence, and age-standardised cancer prevalence—factors that might increase morbidity or mortality from infection—and previous exposure to coronaviruses, a factor that might influence both subsequent transmission probability and mortality outcomes. These factors are known or considered influence IFR.* | 2021 |
| ^2^Cumulative infection rate |  | *cumulative infections were calculated by summing up the total estimated daily infections for each national or subnational location over the entire time period (and also for the shorter time period, Jan 1, 2020, to Oct 15, 2020), and were divided by the 2019 estimated population in each location to get the cumulative infections per capita. Adjusted infections per 1000 people will be used. This rate has been adjusted to seasonality, sltitude, GDP per capita, population density, previous betacoronavirus exposure.* | 2021 |
| Cumulative_estimated_daily_excess_deaths_per_100k | The Economist | *Excess mortality is defined as deaths from all causes during a period, after accounting for expected deaths.14 It estimates how many more people died during the COVID-19 pandemic than would be expected under usual conditions. Excess mortality is a more comprehensive assessment of the pandemic toll than confirmed COVID-19 deaths, as it also captures incorrectly diagnosed or reported deaths and indirect mortality resulting from overburdened healthcare systems or exacerbated poverty. Greater values of excess mortality per 100 000 individuals indicates poor pandemic response.* | 2020; 2021 e 2022 |
| People_fully_vaccinated (cumulative) | Our World in Data - Oxford Martin School | *Total number of people who received all doses prescribed by the initial vaccination protocol* | Last update (February 15th) |
| Delivered population | United Nations | *Delivered vaccines includes vaccines that have been made available in the country (% of population)* | Last update (February 15th) |
| Vaccination policy | Oxford COVID-19 Government Response  Tracker) | *Countries are grouped into six categories: i) No availability; ii) Availability for ONE of following: key workers/ clinically vulnerable groups / elderly groups; iii) Availability for TWO of following: key workers/ clinically vulnerable groups / elderly groups; iv) Availability for ALL of following: key workers/ clinically vulnerable groups / elderly groups; v) Availability for all three plus partial additional availability (select broad groups/ages); vi) Universal availability* | - |
| Administration of the  first dose in the country | Our World in Data | *First vaccine date at the country level* | - |
| The total number of vaccination doses administered per 100 people at the country level (cumulative) | Our World in Data | *Number of vaccine doses administered per 100 people within a given population. All doses, including boosters, are counted individually”.* | Last update (February 15th) |

^1,2^Reference: Bollyky TJ et all. Pandemic preparedness and COVID-19: an exploratory analysis of infection and fatality rates, and contextual factors associated with preparedness in 177 countries, from Jan 1, 2020, to Sept 30, 2021. Lancet 2022; 399: 1489–512. <https://doi.org/10.1016/> S0140-6736(22)00172-6
