# Supplementary material for: The impact of social cohesion and risk communication on excess mortality due to COVID-19 in 213 countries: a retrospective analysis
Source: BMC Public Health. 2024 Jun 14;24:1598. doi: 10.1186/s12889-024-19076-7 (PMC11179214; doi:10.1186/s12889-024-19076-7)
Supplement: Supplementary file 3 — Supplementary Material 3 [file 12889_2024_19076_MOESM3_ESM.docx]

**Supplementary material 3: The basis for the linearity of the independent variables in the linear regression model.**

Added-variable plots” (also called “partial regression plots” or “adjusted variable plots”) are refined residual plots that provide graphic information about the marginal importance of a predictor variable given the other variables already in the model.

The fact that an added-variable plot may suggest “the nature of the functional form” in which a predictor variable should be added to the regression model is more important than that the variable possible inclusion (which can be resolved without graphical help). The added-variable plots play the role that we use scatter diagrams for in simple linear regression; they would tell if data transformation or if certain polynomial model is desirable.

The partial residual (Partial Regressor Residual) plots for the three models indicated that a linear term in any of the independent variables can provide a useful contribution to the regression model, given that the other independent variables are already in the model. Therefore, with the analysis of partial residual graphs, the assumption of linearity can be verified for all independent variables in the multiple linear regression models analyzed (Figure 1, 2 and 3).

| **Parameter Estimates** | | | | | | |
| --- | --- | --- | --- | --- | --- | --- |
| **Variable** | **Label** | **DF** | **Parameter Estimate** | **Standard Error** | **t Value** | **Pr > \|t\|** |
| **Intercept** | Intercept | **1** | 23.26502 | 18.88797 | 1.23 | 0.2219 |
| **Percentual_Population_ages65_abo** | Percentual_Population_ages65_above | **1** | 7.54564 | 1.46786 | 5.14 | <.0001 |
| **Percentual_Estimated_population1** | Percentual_Estimated_population500m_elevation | **1** | -0.50655 | 0.16848 | -3.01 | 0.0036 |
| **High_body_mass_index** | High_body_mass_index | **1** | 3.50782 | 0.67028 | 5.23 | <.0001 |
| **Social__safety_nets_2020** | Social_ safety_nets_2020 | **1** | -14.26407 | 4.89618 | -2.91 | 0.0047 |
| **Unemployment_2019** | Unemployment_2019 | **1** | -1.18521 | 0.58087 | -2.04 | 0.0449 |
| **Covid_related_disorder_events** | Covid_related_disorder_events | **1** | 0.11111 | 0.03426 | 3.24 | 0.0018 |

Table 1: Multiple linear regression models for daily and cumulative excess deaths due to COVID-19 per 100 thousand, in 2020, by sociodemographic, vaccination, health, social cohesion and risk communication variables (n = 81).


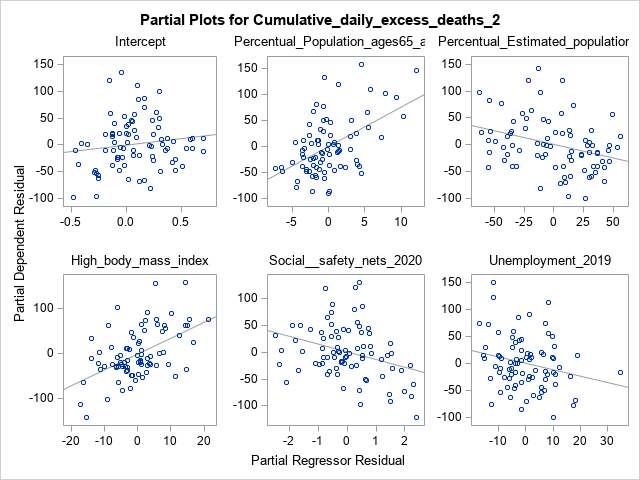


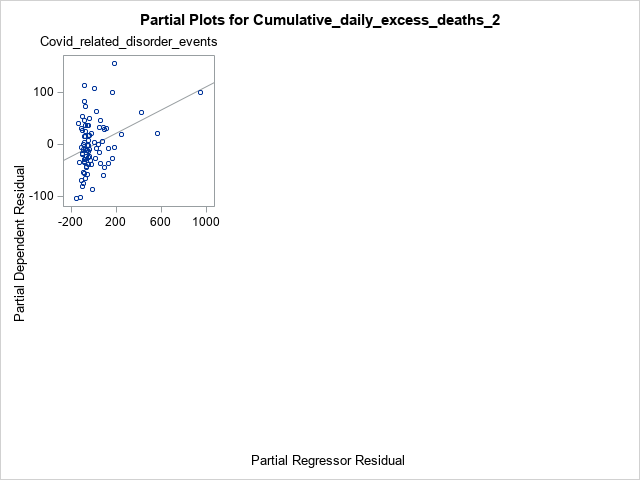


Figure 1: Partial Plots for daily and cumulative excess deaths due to COVID-19 per 100 thousand in 2020.

| **Parameter Estimates** | | | | | | |
| --- | --- | --- | --- | --- | --- | --- |
| **Variable** | **Label** | **DF** | **Parameter Estimate** | **Standard Error** | **t Value** | **Pr > \|t\|** |
| **Intercept** | Intercept | **1** | 17.02687 | 79.74209 | 0.21 | 0.8315 |
| **Percentual_Estimated_population5** | Percentual_Estimated_population5m_elevation | **1** | -3.94060 | 1.96650 | -2.00 | 0.0456 |
| **High_body_mass_index** | High_body_mass_index | **1** | 2.72889 | 1.26371 | 2.16 | 0.0338 |
| **Cardiovascular_diseases_Prevalen** | Cardiovascular_diseases_Prevalence | **1** | 0.04518 | 0.00497 | 9.08 | <.0001 |
| **Social_capital_2020** | Social_capital_2020 | **1** | -29.03553 | 10.36240 | -2.80 | 0.0064 |
| **Public_trust_politicians_2017** | Public_trust_politicians_2017 | **1** | -61.62222 | 17.73300 | -3.48 | 0.0008 |
| **Transparency_government_2017** | Transparency_government_2017 | **1** | 52.97532 | 26.48571 | 2.00 | 0.0417 |

Table 2: Multiple linear regression models for daily and cumulative excess deaths due to COVID-19 per 100 thousand, in 2021, by sociodemographic, vaccination, health, social cohesion and risk communication variables (n = 87).


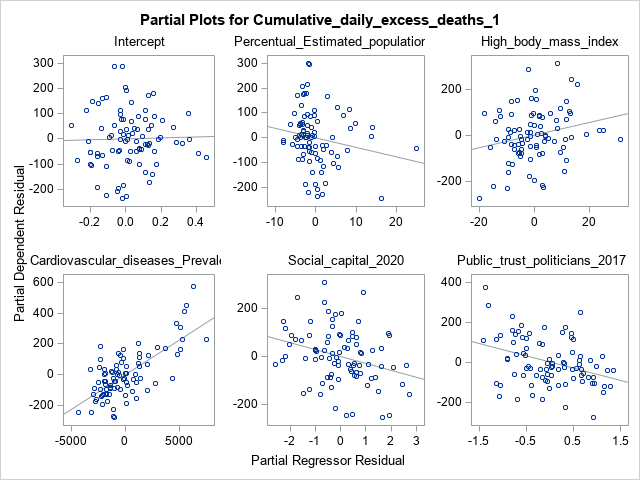


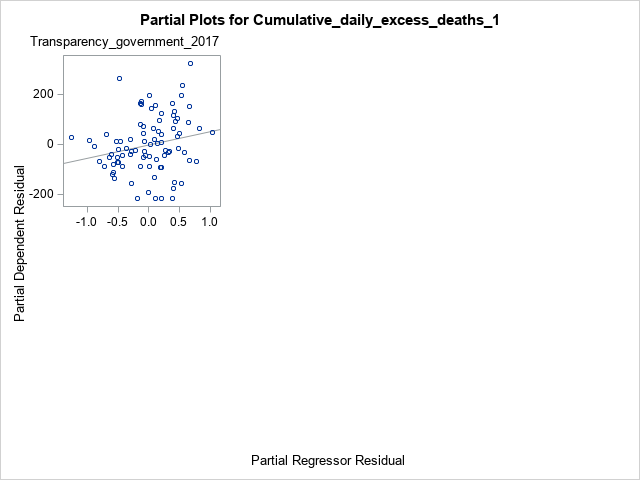


Figure 2: Partial Plots for daily and cumulative excess deaths due to COVID-19 per 100 thousand in 2021.

| **Parameter Estimates** | | | | | | |
| --- | --- | --- | --- | --- | --- | --- |
| **Variable** | **Label** | **DF** | **Parameter Estimate** | **Standard Error** | **t Value** | **Pr > \|t\|** |
| **Intercept** | Intercept | **1** | 70.34529 | 39.16024 | 1.80 | 0.0759 |
| **Percentual_Population_ages65_abo** | Percentual_Population_ages65_above | **1** | 27.35987 | 2.90215 | 9.43 | <.0001 |
| **High_body_mass_index** | High_body_mass_index | **1** | 5.68772 | 1.55840 | 3.65 | 0.0004 |
| **Equal_distribution_resources_ind** | Equal_distribution_resources_index_2019 | **1** | -274.40121 | 69.22511 | -3.96 | 0.0001 |

Table 3: Multiple linear regression models for daily and cumulative excess deaths due to COVID-19 per 100 thousand, in 2022, by sociodemographic, vaccination, health, social cohesion and risk communication variables (n = 92).


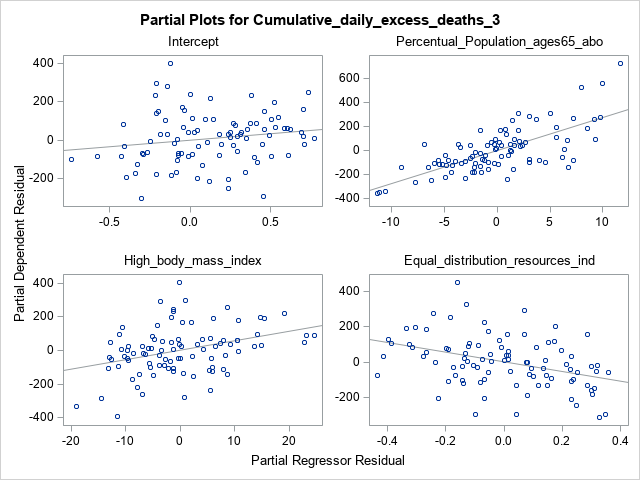


Figure 3: Partial Plots for daily and cumulative excess deaths due to COVID-19 per 100 thousand in 2022.
